# Quantitative assessment of bone mineral density in cervical spine surgery: Diagnostic accuracy of C2 and C3 Hounsfield units versus cervical vertebral bone quality scores

**DOI:** 10.1016/j.xnsj.2026.100923

**Published:** 2026-06-25

**Authors:** Zhan Wang, Qunfei Yu, Wanli Li, Lei Yu, Fangcai Li, Ning Zhang

**Affiliations:** a Department of Orthopedic Surgery, The Second Affiliated Hospital, Zhejiang University School of Medicine, Jiefang Road 88, Hangzhou 310009, Zhejiang, China; b Orthopedics Research Institute of Zhejiang University, Jiefang Road 88, Hangzhou 310009, Zhejiang, China; c Clinical Research Center of Motor System Disease of Zhejiang Province, Jiefang Road 88, Hangzhou 310009, Zhejiang, China; d Zhejiang Key Laboratory of Motor System Disease Precision Research and Therapy, Jiefang Road 88, Hangzhou 310009, Zhejiang, China; e Nursing Department, The Second Affiliated Hospital, Zhejiang University School of Medicine, Jiefang Road 88, Hangzhou 310009, Zhejiang, China; f Department of Radiology, The Second Affiliated Hospital, Zhejiang University School of Medicine, Jiefang Road 88, Hangzhou 310009, Zhejiang, China

**Keywords:** Hounsfield unit, Vertebral bone quality score, Bone mineral density, Osteoporosis, Osteopenia, Cervical spine surgery

## Abstract

**Background:**

Preoperative bone mineral density (BMD) assessment is essential for cervical spine surgery, yet dual-energy X-ray absorptiometry (DXA) is not routinely performed. Opportunistic imaging metrics such as computed tomography-derived Hounsfield units (HU) and magnetic resonance imaging-based vertebral bone quality (VBQ) scores have been proposed as surrogates, but their comparative diagnostic value in the cervical spine remains unclear.

**Methods:**

In this retrospective single-center study, 154 patients aged ≥50 years undergoing cervical spine surgery for degenerative diseases were included. All patients underwent cervical CT, cervical MRI, DXA, and bone turnover marker testing within 3 months preoperatively. HU values at C2–C7 were measured on sagittal CT images, and VBQ scores were calculated from T1-weighted MRI. Correlations between imaging parameters and DXA T-scores (lumbar spine, femoral neck, and total hip) were analyzed. Receiver operating characteristic analysis evaluated diagnostic performance for reduced BMD (T-score <−1.0), including sex-specific subgroups.

**Results:**

Mean cervical HU values decreased progressively from C4 to C7 and were significantly lower in patients with reduced BMD (all p < .001). C2 HU showed the strongest correlations with DXA T-scores (r = 0.528–0.671). C2 and C3 HU demonstrated numerically higher discriminative ability for identifying reduced BMD (area under the curve [AUC] = 0.737 and 0.741) compared with VBQ (AUC = 0.638) and Goutallier grade (AUC = 0.623). Performance of C2 HU was consistent across sexes. HU values showed weak correlations with β-cross-linked telopeptide of type I collagen and osteocalcin and no association with 25-hydroxyvitamin D or procollagen type I N-terminal propeptide.

**Conclusions:**

Upper cervical HU measurements, particularly at C2 and C3, demonstrated favorable discriminative performance for identifying reduced BMD and may serve as a practical opportunistic screening tool in patients undergoing cervical spine surgery.

## Introduction

Quantitative assessment of bone mineral density (BMD) in cervical spine surgery has gained attention due to its clinical relevance for surgical outcomes. Osteoporotic patients were more likely to undergo revision surgery, have longer hospitalizations, and have higher hospitalization costs, than their nonosteoporotic counterparts [1]. Furthermore, osteoporosis is consistently listed as an exclusion criterion in investigational device exemption trials for cervical disc replacement, underscoring its clinical importance [2]. While dual-energy X-ray absorptiometry (DXA) screening is the current method of assessing BMD, the majority of patients do not have DXA measurements available before undergoing surgical instrumentation.

Recently, a simple Magnetic resonance imaging (MRI)-based scoring system was used to evaluate the lumbar spine’s vertebral bone quality (VBQ). Ehresman et al. [3] first used noncontrast T1-weighted MRIs of the lumbar spine to create VBQ score and found the novel VBQ score is a significant predictor of osteopenia/osteoporosis with an accuracy of 81%. Subsequently, Soliman et al. [4] developed the cervical VBQ (C-VBQ) based on the lumbar VBQ score, which has shown significant correlation with the lumbar VBQ and demonstrated predictive value for postoperative cage subsidence in cervical spine surgery patients. Recently, Huang et al. [5] revealed that cervical VBQ scores were significantly correlated with DXA T-score for patients undergoing cervical spine surgery, achieving good discriminative performance for identifying patients with reduced BMD, offering a radiation-free screening alternative. However, Oezel et al. [6] found weak to moderate negative correlations between VBQ and Quantitative Computed Tomography (QCT) measurements in 102 cervical surgery patients, suggesting limited clinical utility. Therefore, the above research indicates that the value of VBQ in evaluating bone quality during cervical spine surgery requires further study to clarify.

Several studies using lumbar Computed tomography（CT )scans have demonstrated a positive correlation between HU values and BMD measured by DXA [[7], [8], [9]]. More recently, HU values derived from cervical CT have also shown significant correlations with DXA T-scores, suggesting their potential utility in assessing cervical bone quality [10,11]. Because patients undergoing cervical spine surgery commonly receive both cervical CT and MRI during routine preoperative evaluation, direct comparison of CT-based and MRI-based opportunistic bone quality metrics is clinically feasible and may help identify the most practical screening strategy. Furthermore, this study aligns with the growing concept of opportunistic imaging, which seeks to extract clinically relevant information from routinely acquired imaging without additional cost, radiation, or patient burden [12,13]. However, it remains unclear whether CT-derived HU measurements provide better discriminative performance than MRI-based C-VBQ scores for identifying reduced BMD in patients undergoing cervical spine surgery. In addition, the optimal cervical vertebral level for prediction and the corresponding site-specific predictive equations have not yet been established.

## Material and methods

### Study population

**Fig. 1:**
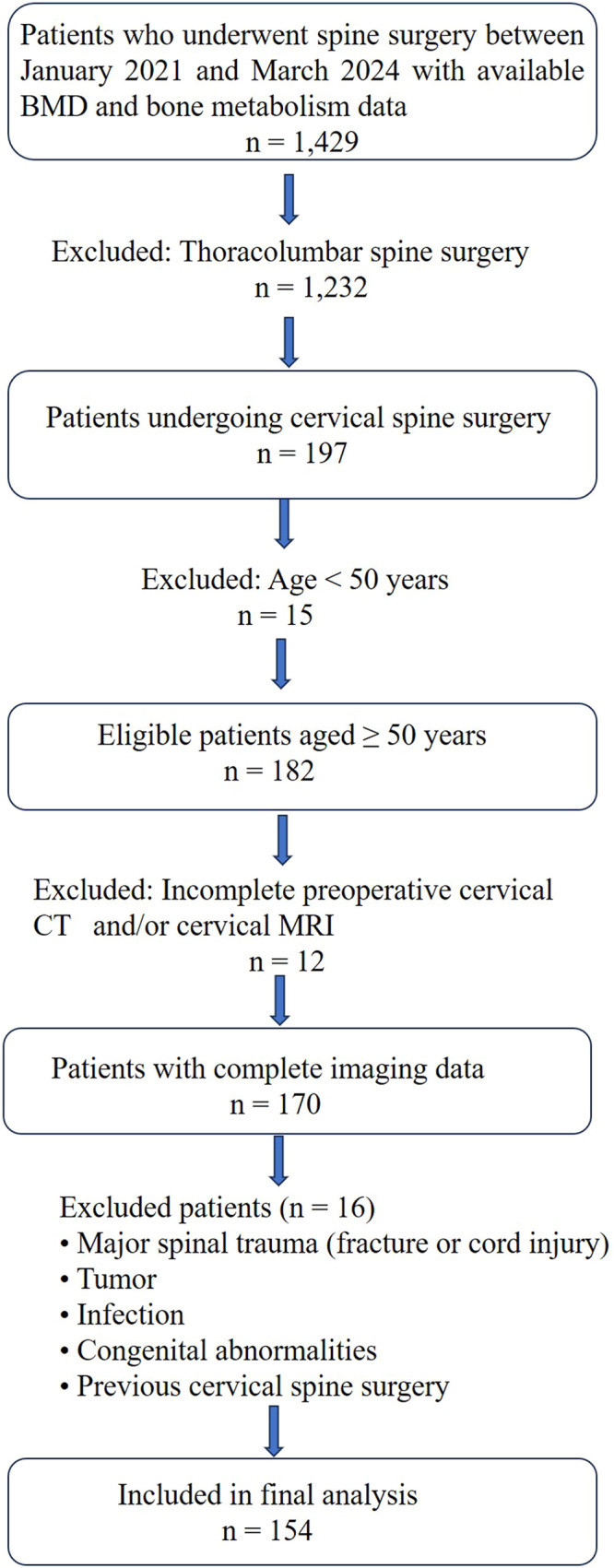
Flowchart of patient selection. BMD, bone mineral density.

Institutional Review Board approval (No. 2024-0241) was obtained and all procedures were conducted in accordance with the Declaration of Helsinki. The informed consent was waived by the institutional review board because of the retrospective study design. We retrospectively reviewed the medical and radiological records of patients who underwent cervical spine surgery for degenerative conditions between January 2021 and March 2024 at a single tertiary referral center. All patients underwent cervical CT, cervical MRI, DXA, and bone turnover marker (BTM) testing within 3 months before surgery. Patients younger than 50 years were excluded. Additional exclusion criteria included major spinal trauma (fracture or cord injury), tumor, infection, congenital abnormalities, and a history of cervical spine surgery (Fig. 1).

Demographic data were collected, including age, sex, body mass index (BMI), smoking status, alcoholism, hyperlipidemia, and diabetes. DXA measurements included T-scores of the femoral neck, total hip, and lumbar spine (L1–L4). According to World Health Organization criteria, osteoporosis is defined as a T-score of −2.5 or lower, osteopenia as a T-score between −1.0. and −2.5, and normal bone mass as a T-score of −1.0 or higher. For the primary diagnostic analysis, reduced BMD was defined as a T-score <−1.0 using the lowest value obtained from the lumbar spine, femoral neck, or total hip. BTMs included serum 25-hydroxyvitamin D [25(OH)D], β-cross-linked telopeptide of type I collagen (β-CTX), procollagen type I N-terminal propeptide (PINP), and osteocalcin (OC).

### Imaging evaluation

**Fig. 2:**
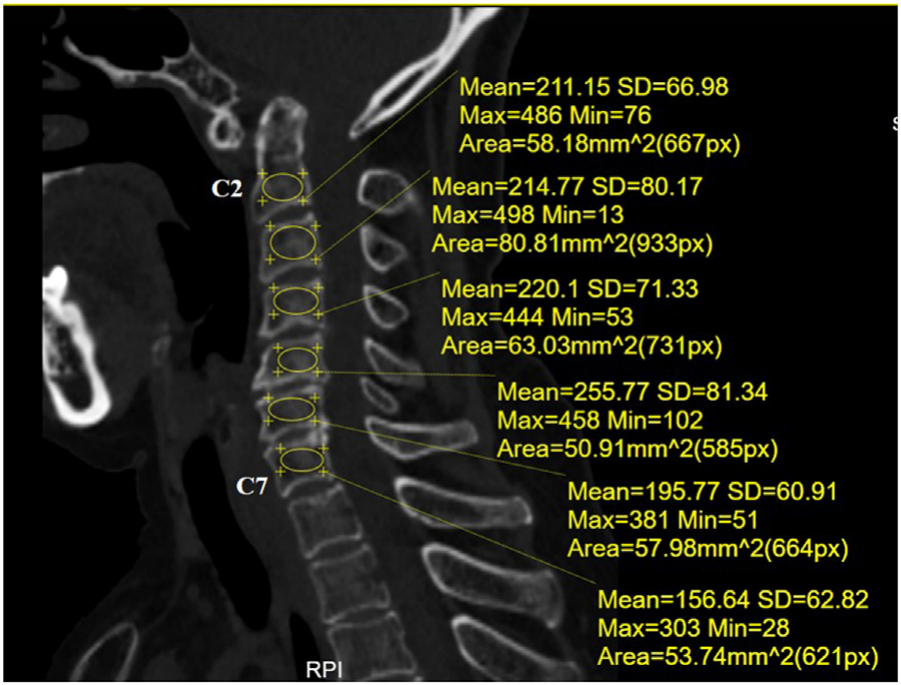
Illustration of Hounsfield unit (HU) measurements from C2 to C7 on a sagittal CT image.

HU values of the C2–C7 vertebrae were measured on midsagittal CT images by drawing an elliptical region of interest (ROI) within the trabecular portion of each vertebral body, carefully excluding all cortical margins such as the endplates and anterior–posterior cortical walls [11] (Fig. 2). Cervical CT examinations were performed using a multidetector CT scanner. Scans were obtained with a tube voltage of 120 kVp and automatic tube current modulation. Images were reconstructed with a standard bone algorithm at a slice thickness of 1.0 mm. All CT scans used for HU measurements were noncontrast studies.

The C-VBQ score was calculated from midsagittal T1-weighted noncontrast MRI images obtained using a 3.0-T scanner, following the previously described method [4]. ROIs were placed within the medullary bone of the C3–C6 vertebral bodies, and an additional ROI was placed in the cerebrospinal fluid at the C2 level; if C2 was obstructed, the cerebrospinal fluid at C1 was used. The signal intensity (SI) was first measured individually at each vertebral level (C3–C6), and the median SI was then divided by the cerebrospinal fluid SI.

Cervical paraspinal fatty infiltration (FI) was visually graded on axial T2-weighted MRI using the Goutallier classification for the bilateral semispinalis cervicis muscles at the C5–C6 level. Grades were defined as follows: Grade 0, no FI; Grade 1, minimal FI; Grade 2, more muscle than fat; Grade 3, equal amounts of fat and muscle; and Grade 4, more fat than muscle. Patients were subsequently categorized as having mild (Grades 0–1), moderate (Grade 2), or severe paraspinal fatty infiltration (Grades 3–4).

### Statistical analysis

Continuous variables were presented as mean ± standard deviation (SD), and categorical variables as percentages. The Shapiro–Wilk test was used to assess the normality of continuous variables. Categorical variables were compared using the chi-square test, whereas continuous variables were compared using the independent-samples t-test or the Mann–Whitney U test, depending on distribution.

Receiver operating characteristic (ROC) curves were generated to evaluate the performance of imaging parameters in identifying reduced BMD (T-score <−1.0). The area under the ROC curve (AUC) with corresponding 95% confidence intervals (CIs) was calculated for each parameter. Optimal cutoff values were determined using the Youden index. Sensitivity, specificity, positive predictive value (PPV), and negative predictive value (NPV) were subsequently calculated for the optimal cutoff values.

Pearson correlation analysis was performed to assess the relationships between cervical HU values, DXA T-scores, and BTMs. Site-specific linear regression equations between C2 HU and DXA T-scores were derived from the fitted correlation lines. Model fit was assessed using the coefficient of determination (R^2^), and residual plots were examined to evaluate regression assumptions. Internal validation of the regression models was performed using bootstrap resampling with 1,000 iterations, and bias-corrected accelerated (BCa) 95% confidence intervals were calculated.

Two independent observers blinded to DXA results performed HU and C-VBQ measurements. Interobserver and intraobserver reliability were assessed using intraclass correlation coefficients (ICCs). Interobserver reliability was excellent for HU measurements (ICC = 0.94) and good for C-VBQ measurements (ICC = 0.88). Intraobserver reliability was similarly high for HU (ICC = 0.96) and C-VBQ measurements (ICC = 0.91).

All statistical analyses were performed using SPSS version 27.0 (SPSS Inc.). A p-value <.05 was considered statistically significant.

## Results

### Patient characteristics

A total of 154 patients were included, comprising 80 with normal BMD, 53 with osteopenia, and 21 with osteoporosis. The mean age was significantly higher in the osteoporosis group (69.48 ± 7.26 years) compared with the osteopenia (62.92 ± 8.72 years) and normal groups (63.31 ± 8.88 years; p = .009). Female sex predominated in the osteoporosis group (95.2%), whereas males comprised the majority in the normal group (61.3%; p < .001). No significant differences were found among groups for BMI, smoking, alcoholism, hyperlipidemia, or diabetes (all p > .05) (Table 1).

**Table 1:** Baseline demographic and clinical characteristics of patients undergoing cervical spine surgery (N = 154).

	Normal (n = 80)	Osteopenia (n = 53)	Osteoporosis (n = 21)	p
Age	63.31 ± 8.88	62.92 ± 8.72	69.48 ± 7.26	.009
Sex				
Male	49 (61.3%)	23 (43.4%)	1 (4.8%)	<.001
Female	31 (38.8%)	30 (56.6%)	20 (95.2%)	
BMI	23.79 ± 2.76	22.95 ± 2.87	22.38 ± 4.29	.097
Smoking				.464
No	63 (78.8%)	41 (77.4%)	19 (90.5%)	
Yes	17 (21.3%)	12 (22.6%)	2 (9.5%)	
Alcoholism				.925
No	73 (91.3%)	48 (90.6%)	20 (95.2%)	
Yes	7 (8.8%)	5 (9.4%)	1 (4.8%)	
Hyperlipidemia				.819
No	47 (58.8%)	34 (64.2%)	13 (61.9%)	
Yes	33 (41.3%)	19 (35.8%)	8 (38.1%)	
Diabetes				1
No	64 (80.0%)	42 (79.2%)	17 (81.0%)	
Yes	16 (20.0%)	11 (20.8%)	4 (19.0%)	
C2 HU	360.98 ± 87.99	302.50 ± 72.17	245.76 ± 72.49	<.001
C3 HU	344.62 ± 76.80	296.56 ± 60.39	245.51 ± 51.11	<.001
C4 HU	357.99 ± 74.57	309.35 ± 70.45	232.11 ± 56.20	<.001
C5 HU	342.30 ± 89.95	289.90 ± 73.46	220.89 ± 58.09	<.001
C6 HU	317.72 ± 107.98	258.53 ± 70.11	202.94 ± 61.77	<.001
C7 HU	273.46 ± 70.06	232.16 ± 51.94	196.39 ± 49.44	<.001
C VBQ	3.11 ± 0.90	3.52 ± 1.52	3.71 ± 0.86	.037
Goutallier grade (C5–C6)				.027
0–1	54 (67.5%)	25 (47.2%)	7 (33.3%)	
2	16 (20.0%)	17 (32.1%)	8 (38.1%)	
3–4	10 (12.5%)	11 (20.8%)	6 (28.6%)	

BMI, body mass index; HU, Hounsfield units; VBQ, vertebral bone quality.

### Cervical HU and VBQ measurements

Mean HU values decreased progressively from C4 to C7 in all groups and were significantly lower in osteoporotic patients compared with the other groups (p < .001 for all levels). For example, C2 HU values were 360.98 ± 87.99 in the normal group, 302.50 ± 72.17 in the osteopenia group, and 245.76 ± 72.49 in the osteoporosis group. VBQ scores were significantly higher in the osteoporosis group (3.71 ± 0.86) compared with the normal group (3.11 ± 0.90; p = .037). Goutallier grade at C5–C6 was also associated with BMD status (p = .027), with higher grades more prevalent in osteoporotic patients (Table 1).

### Diagnostic performance

**Fig. 3:**
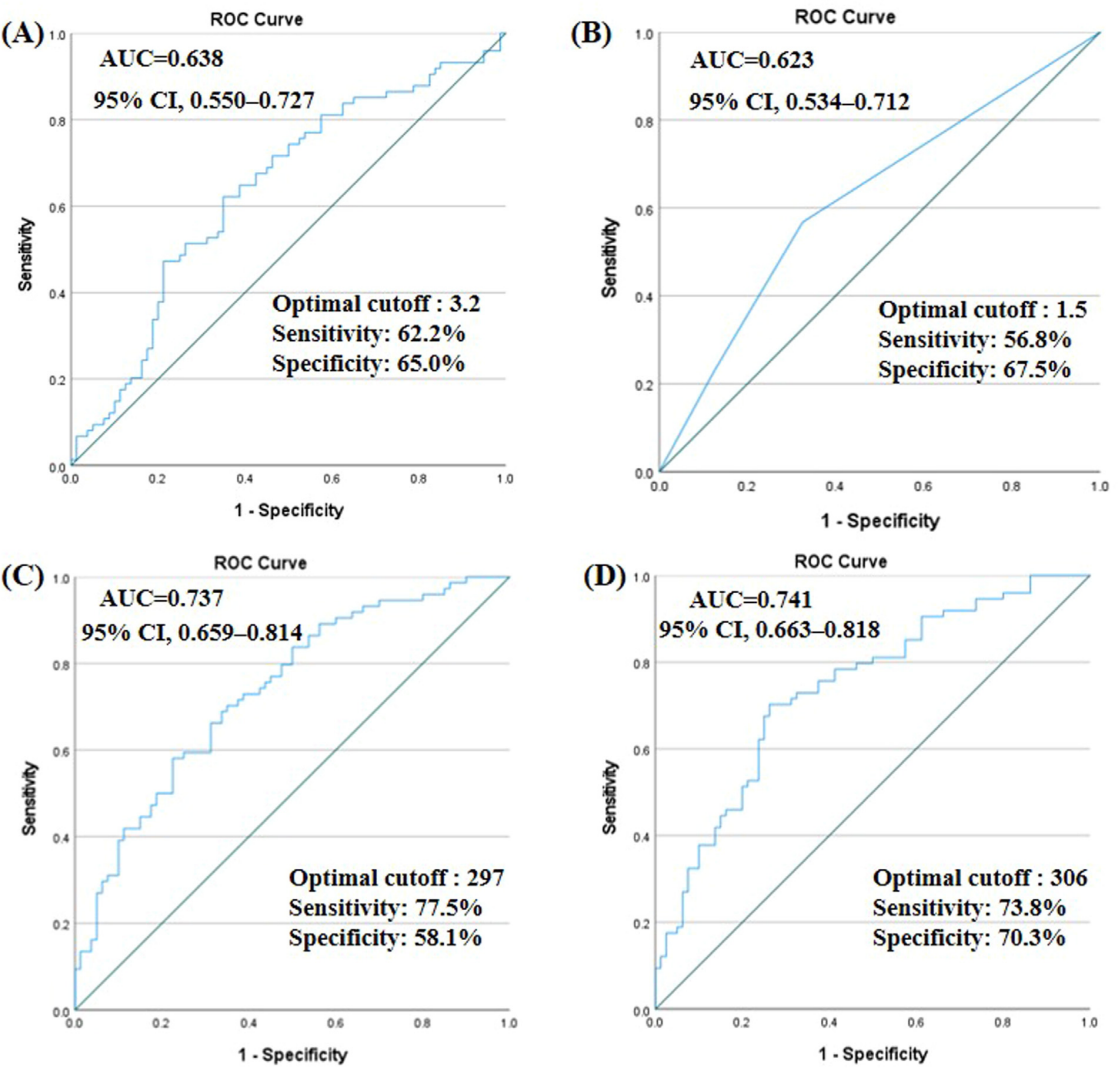
Receiver operating characteristic (ROC) curves illustrating the predictive performance of various variables for identifying reduced bone mineral density (T-score <−1.0). (A) Vertebral bone quality (VBQ); (B) Goutallier grade at C5–C6; (C) Hounsfield unit (HU) value of C2 vertebra; (D) HU value of C3 vertebra. AUC, area under the curve; CI, confidence interval.

ROC analysis demonstrated that C2 HU and C3 HU provided favorable discriminative performance for identifying reduced BMD, with AUC values of 0.737 (95% CI 0.659–0.814) and 0.741 (95% CI 0.663–0.818), respectively. The optimal cutoff values determined by the Youden index were 297 for C2 HU and 306 for C3 HU, yielding sensitivities of 77.5% and 73.8% and specificities of 58.1% and 70.3%, respectively (Fig. 3). For C2 HU, the PPV and NPV were 69.4% and 66.3%, respectively. For C3 HU, the PPV and NPV were 70.3% and 72.5%, respectively.

In contrast, the VBQ score and Goutallier grade showed lower diagnostic accuracy, with AUC values of 0.638 (95% CI 0.550–0.727) and 0.623 (95% CI 0.534–0.712), respectively (Fig. 3). The optimal cutoff values were 3.2 for the VBQ score and 1.5 for the Goutallier grade, corresponding to sensitivities of 62.2% and 56.8% and specificities of 65.0% and 67.5%, respectively.

### Correlation between HU values and DXA T-scores

Across all patients, cervical HU values showed moderate-to-strong positive correlations with DXA T-scores at the lumbar spine, femoral neck, and total hip (all p < .001). C2 HU values demonstrated the strongest correlations, with coefficients ranging from r = 0.528 for the femoral neck to r = 0.671 for the L1–L4 average T-score (Table 2). Similar trends were observed in both sexes. In both male and female patients, C2 and C3 HU consistently demonstrated strong predictive value for T-scores across all anatomical sites, maintaining the highest or near-highest correlation coefficients among cervical vertebrae within each sex (Table 3, Table 4).

**Table 2:** Correlation between cervical vertebral HU and DXA T-scores.

	C2 HU	C3 HU	C4 HU	C5 HU	C6 HU	C7 HU
L1 T value	0.658*	0.658*	0.601*	0.592*	0.503*	0.503*
L2 T value	0.631*	0.636*	0.604*	0.545*	0.460*	0.443*
L3 T value	0.631*	0.615*	0.589*	0.559*	0.471*	0.471*
L4 T value	0.619*	0.570*	0.547*	0.535*	0.461*	0.475*
L1-L4 T value	0.671*	0.651*	0.615*	0.598*	0.517*	0.514*
Femoral neck T value	0.528*	0.531*	0.533*	0.498*	0.481*	0.499*
Hip T value	0.575*	0.564*	0.582*	0.550*	0.502*	0.543⁎⁎

DXA, dual-energy X-ray absorptiometry; HU, Hounsfield units.

⁎ Signifies that correlation is significant at the 0.001 level (2-tailed).

**Table 3:** Correlation between cervical vertebral HU and DXA T-scores in female patients.

	C2 HU	C3 HU	C4 HU	C5 HU	C6 HU	C7 HU
L1 T value	0.651*	0.701*	0.573*	0.573*	0.520*	0.565*
L2 T value	0.656*	0.716*	0.640*	0.597*	0.547*	0.545*
L3 T value	0.630*	0.665*	0.607*	0.601*	0.515*	0.579*
L4 T value	0.559*	0.544*	0.479*	0.500*	0.424*	0.490*
L1-L4 T value	0.667*	0.694*	0.609*	0.613*	0.528*	0.577*
Femoral neck T value	0.558*	0.555*	0.533*	0.505*	0.503*	0.530*
Hip T value	0.560*	0.558*	0.546*	0.508*	0.483*	0.535⁎⁎

DXA, dual-energy X-ray absorptiometry; HU, Hounsfield units.

⁎ Signifies that correlation is significant at the 0.001 level (2-tailed).

**Table 4:** Correlation between cervical vertebral HU and DXA T-scores in male patients.

	C2 HU	C3 HU	C4 HU	C5 HU	C6 HU	C7 HU
L1 T value	0.617*	0.612*	0.578*	0.586*	0.508*	0.474*
L2 T value	0.543*	0.543*	0.523*	0.470*	0.378*	0.361*
L3 T value	0.571*	0.554*	0.528*	0.511*	0.451*	0.411*
L4 T value	0.601*	0.547*	0.542*	0.531*	0.496*	0.466*
L1-L4 T value	0.627*	0.606*	0.586*	0.584*	0.533*	0.493*
Femoral neck T value	0.418*	0.454*	0.476*	0.460*	0.424*	0.458*
Hip T value	0.507*	0.521*	0.570*	0.583*	0.517*	0.578*

DXA, dual-energy X-ray absorptiometry; HU, Hounsfield units.

⁎ Signifies that correlation is significant at the 0.001 level (2-tailed).

**Fig. 4:**
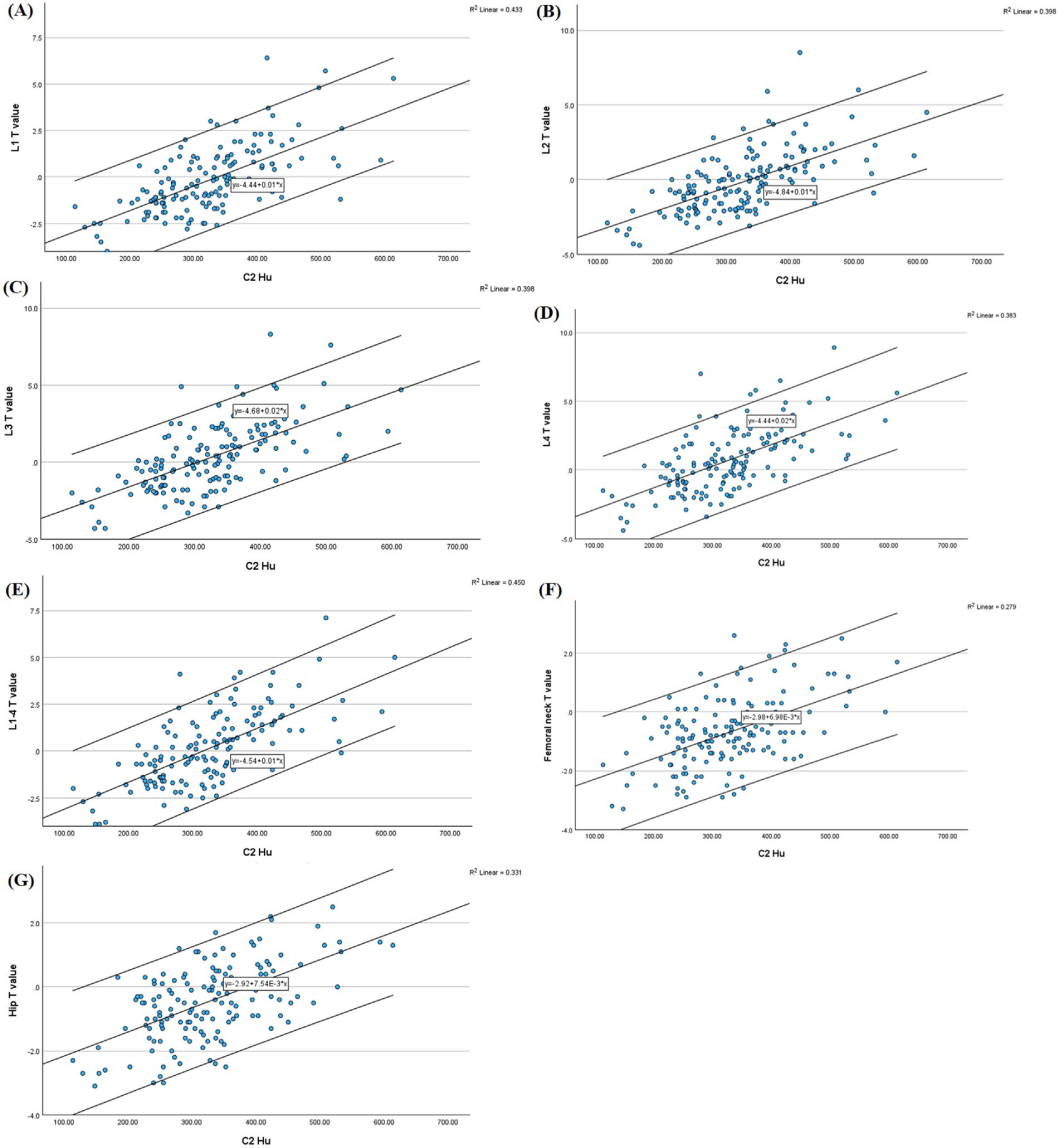
Pearson correlation plots between Hounsfield units (HU) of the C2 vertebral body and dual-energy X-ray absorptiometry (DXA) T-scores at various anatomical sites: (A) L1, (B) L2, (C) L3, (D) L4, (E) L1–L4 average, (F) femoral neck, and (G) total hip.

**Fig. 5:**
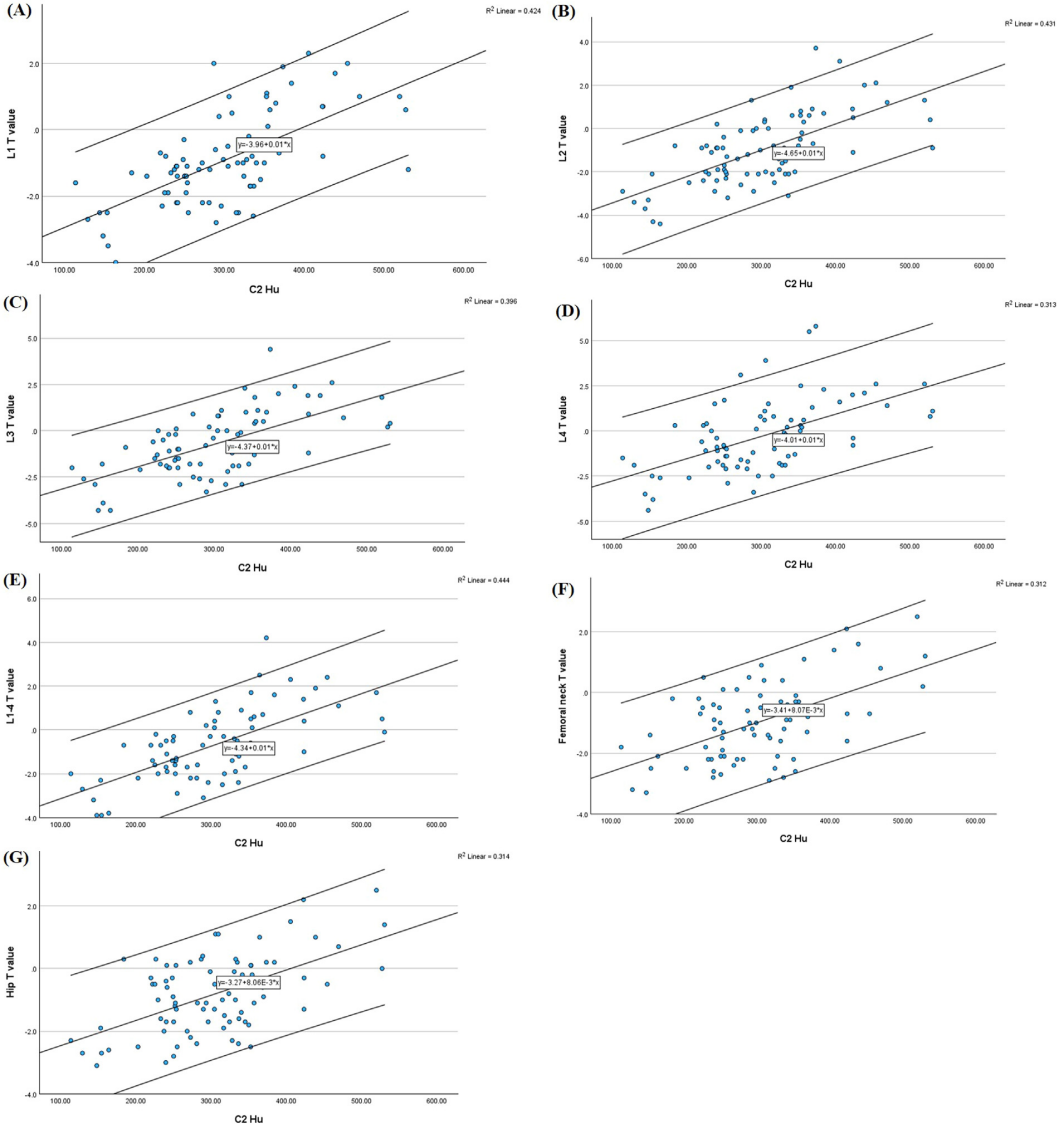
Pearson correlation plots between Hounsfield units (HU) of the C2 vertebral body and dual-energy X-ray absorptiometry (DXA) T-scores at various anatomical sites in female patients: (A) L1, (B) L2, (C) L3, (D) L4, (E) L1–L4 average, (F) femoral neck, and (G) total hip.

**Fig. 6:**
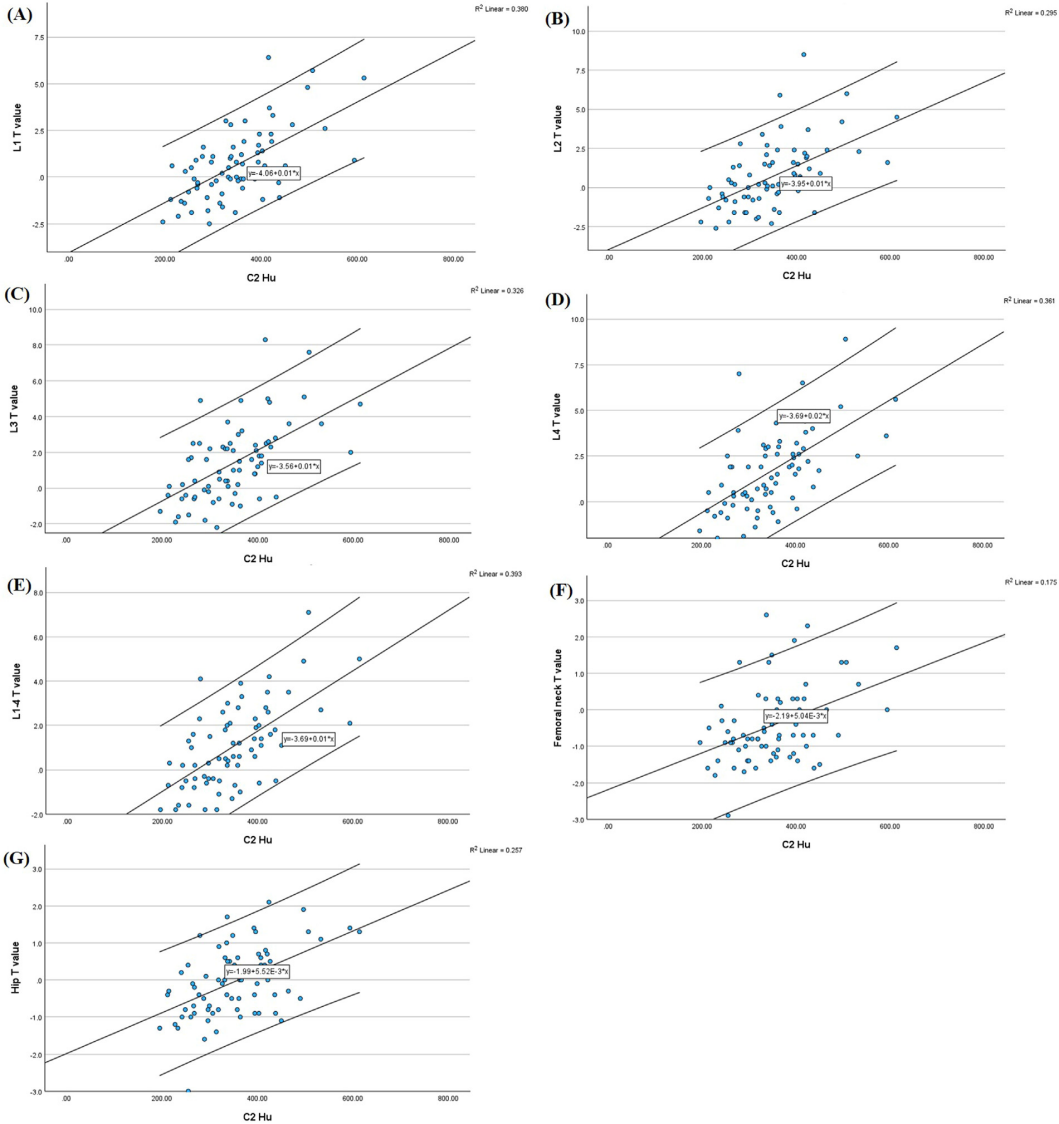
Pearson correlation plots between Hounsfield units (HU) of the C2 vertebral body and dual-energy X-ray absorptiometry (DXA) T-scores at various anatomical sites in male patients: (A) L1, (B) L2, (C) L3, (D) L4, (E) L1–L4 average, (F) femoral neck, and (G) total hip.

As illustrated in Figs. 4 to 6, scatter plots further confirmed the positive linear relationship between C2 HU and DXA T-scores at various skeletal sites in the overall cohort (Fig. 4), female subgroup (Fig. 5), and male subgroup (Fig. 6). Each Pearson correlation plot included a fitted linear regression line with the corresponding equation, providing site-specific relationships between C2 HU and DXA T-scores, all of which demonstrated a good fit across subgroups. Visual inspection of residual plots and normal probability plots demonstrated no major violations of linear regression assumptions.

Bootstrap validation using 1,000 resamples demonstrated stable regression coefficients across all skeletal sites illustrated in Figs. 4 to 6. For lumbar spine T-scores, the regression coefficients remained significant at L1 (β = 0.013, 95% CI 0.010–0.016), L2 (β = 0.014, 95% CI 0.011–0.018), L3 (β = 0.015, 95% CI 0.013–0.019), L4 (β = 0.016, 95% CI 0.013–0.019), and the combined L1–L4 measurement (β = 0.014, 95% CI 0.011–0.017). Similarly, significant associations were observed for femoral neck T-scores (β = 0.007, 95% CI 0.005–0.009) and total hip T-scores (β = 0.008, 95% CI 0.006–0.009). The bootstrap-derived confidence intervals did not cross zero, supporting the robustness and stability of the regression models.

### Correlation between HU values and BTMs

Cervical HU values exhibited weak negative correlations with β-CTX and osteocalcin, reaching statistical significance for C2 HU with β-CTX (r = −0.204, p < .05) and OC (r = −0.163, p < .05). No significant associations were found between cervical HU values and 25(OH)D or PINP levels (all p > .05) (Table 5).

**Table 5:** Correlation relationship of cervical CT value and bone turnover markers.

	C2 Hu	C3 Hu	C4 Hu	C5 Hu	C6 Hu	C7 Hu
25(OH)D, ng/mL	−0.052	−0.078	−0.043	−0.07	0.02	−0.01
β-CTX, ug/mL	−0.204*	−0.129	−0.108	−0.102	−0.131	−0.137
PINP, ng/mL	−0.144	−0.07	−0.003	−0.019	−0.059	−0.099
OC, ng/mL	−0.163*	−0.099	−0.06	−0.046	−0.081	−0.09

25(OH)D, 25-hydroxyvitamin D; β-CTX, β-cross-linked telopeptide of type I collagen; OC, osteocalcin; PINP, procollagen type I N-terminal propeptide.

⁎ Signifies that correlation is significant at the 0.05 level (2-tailed).

## Discussion

In this study we found that cervical CT derived HU measurements, particularly at C2 and C3, correlated moderately to strongly with DXA T-scores across multiple skeletal sites and demonstrated numerically higher discriminative performance than MRI-based C-VBQ and Goutallier grading for identifying reduced BMD. C2 HU and C3 HU achieved the highest AUC values in the ROC analysis, and site specific linear regression models between C2 HU and DXA T-scores showed good fit across subgroups. DXA is widely used to assess preoperative bone quality, yet a survey of more than 100 spine surgeons reported that only 44 percent of patients routinely underwent DXA before surgery [14]. Given this gap in clinical practice, our findings suggest that opportunistic HU measurement on routine cervical CT offers a practical and efficient alternative for screening reduced systemic bone mass in candidates for cervical spine surgery.

Several factors may explain why C2 and C3 HU demonstrated the strongest and most consistent correlations with DXA T-scores. The upper cervical vertebrae are less affected by degenerative changes such as endplate sclerosis, osteophyte formation, and load-related remodeling [11,15], which helps preserve the trabecular attenuation signal and reduces measurement bias. Han et al. [11] also reported that HU values obtained from the upper cervical levels (C2 and C3) more reliably reflected overall BMD than values from lower segments. They further noted that cervical HU provided consistent information regardless of measurement plane, age, sex, or the degree of degeneration. However, their work did not provide specific BMD prediction formulas and did not compare the relative predictive performance of CT HU, C-VBQ, and paraspinal muscle quality. In our cohort, we observed a progressive decline in mean HU values from C2 to C7, along with significantly lower HU values at all levels in patients with osteoporosis, a pattern consistent with previous findings [16]. This supports the presence of a rostral-to-caudal gradient in trabecular structure or disease involvement, which positions C2 and C3 as a relatively preserved and reliable reference level for assessing bone quality on midsagittal CT. These features reinforce the utility of C2 and C3 HU as a practical and convenient index for opportunistic screening. Although Lovecchio et al. [16] recommended incorporating level-specific bone density considerations into cervical spine surgical planning, they did not provide concrete criteria or quantitative tools to guide such assessment. In contrast, our study establishes site-specific prediction equations for estimating bone density in both men and women, offering a clinically applicable framework that may enhance preoperative evaluation and improve overall efficiency.

Recently, a meta-analysis demonstrated that the MRI-based VBQ score has meaningful diagnostic value for identifying osteoporosis and may serve as an opportunistic screening tool before spine surgery [17]. Pu et al. [18] reported that the VBQ score provides a useful preoperative assessment of bone density in patients undergoing lumbar spine procedures, with ROC analysis showing good discrimination for reduced BMD and osteoporosis. In addition, VBQ has been shown to reflect both bone and paravertebral muscle quality, with muscle quality contributing more to the VBQ signal than bone density [19]. Huang et al. [5] were the first to apply the VBQ method in patients undergoing cervical spine surgery and found a significant correlation between C-VBQ scores and DXA T-scores, with an AUC of 0.78. In contrast to these findings, MRI-based parameters in our cohort, including C-VBQ and Goutallier grading, demonstrated weaker performance in predicting reduced BMD, with AUC values of 0.638 and 0.623, respectively. This is consistent with the results of Oezel et al. [6], who reported that VBQ may be insufficient for reliably estimating BMD in the cervical spine. Similarly, Shi et al. [20] found that C-VBQ lost its reliability as an indicator of DXA-derived BMD in obese patients. However, these findings should not be interpreted as indicating an intrinsic limitation of MRI-based bone quality assessment. The lower AUC observed in the present cohort may reflect differences in patient selection, MRI acquisition parameters, imaging protocols, and the distribution of BMD compared with previous studies. Furthermore, C-VBQ may provide complementary rather than inferior clinical information, as previous studies have demonstrated its value in predicting postoperative complications, including cage subsidence [21] and screw loosening [22] following cervical spine surgery. Therefore, the clinical utility of C-VBQ should not be judged solely on its correlation with DXA-derived T-scores. Future research may further clarify the role of C-VBQ in postoperative complications and long-term prognostic outcomes in cervical spine patients.

The relatively weak association between Goutallier grading and BMD observed in the present study should be interpreted with caution. Importantly, Goutallier grading reflects paraspinal fatty infiltration and muscle degeneration rather than a validated diagnosis of sarcopenia, which is formally defined by the presence of reduced muscle mass together with impaired muscle strength and/or physical performance [23,24]. Nevertheless, the biological relationship between muscle degeneration and bone loss remains well recognized within the framework of osteosarcopenia, in which skeletal muscle and bone interact through shared mechanical, metabolic, and endocrine pathways [25]. The relatively modest correlation identified in our cohort may reflect the heterogeneous age distribution, sex imbalance, and multifactorial determinants of both muscle quality and bone health. Accordingly, while Goutallier grading may provide complementary information regarding musculoskeletal degeneration, its utility as a surrogate marker of reduced BMD appears limited compared with direct vertebral imaging parameters such as HU measurements.

Recently, Li et al. [26] were the first to examine the relationship between VBQ scores and BTMs in patients undergoing lumbar spine surgery. They reported that preoperative VBQ assessment on MRI could offer an initial, radiation-free evaluation of bone metabolism and might help guide postoperative osteoporosis management. Notably, VBQ demonstrated stronger correlations with BTMs than HU values and DXA T-scores. In our study, cervical HU values showed only weak negative correlations with selected BTMs, including beta CTX and osteocalcin, and no significant association with 25 hydroxyvitamin D or PINP. These findings should be interpreted as exploratory and hypothesis-generating because BTMs were obtained at a single time point without fasting or timing standardization. The modest strength of these relationships suggests that static imaging markers of trabecular attenuation primarily capture long-term bone mass rather than short-term metabolic dynamics. BTMs, on the other hand, are influenced by circadian rhythm, recent fractures, nutritional status, and medication use [[27], [28], [29]], which can limit their ability to correlate consistently with HU values acquired at a single time point. Despite these limitations, integrating opportunistic HU assessment with targeted laboratory testing may still enhance clinical evaluation. A low cervical HU value detected on routine CT can prompt confirmatory DXA testing, referral for bone health optimization, or adjustments in surgical planning, such as the selection of augmented fixation strategies in patients at risk of poor bone quality.

From a clinical perspective, the present findings support the potential role of opportunistic cervical HU measurements as a screening tool for identifying patients who may benefit from further bone health evaluation before cervical spine surgery. However, the current study focused primarily on the association between imaging biomarkers and DXA-derived BMD rather than postoperative outcomes. Therefore, the present study should be viewed as a necessary precursor to outcome-based validation studies. Although upper cervical HU measurements demonstrated favorable discriminative performance for identifying reduced BMD, further investigation is needed to determine whether C2 and C3 HU independently predict clinically relevant complications such as screw loosening, cage subsidence, instrumentation failure, or revision surgery. Compared with the relatively mature lumbar literature [30,31], outcome-based evidence linking cervical HU measurements to postoperative complications remains limited. Future studies incorporating automated image analysis and larger multicenter cohorts may enable more rigorous comparative assessments among opportunistic imaging biomarkers while improving statistical power for adjusted model development. In addition, outcome-based validation studies are needed to determine whether these imaging parameters independently predict clinically relevant complications and can be integrated into robust predictive models for individualized surgical decision-making.

This study has several limitations. First, the retrospective design from a single tertiary referral center introduces the possibility of selection bias toward older or more symptomatic patients, which may limit generalizability. Second, DXA was used as the reference standard for BMD assessment; however, degenerative lumbar changes such as osteophytes, endplate sclerosis, and facet arthropathy may artificially elevate lumbar spine T-scores in elderly patients, potentially leading to underestimation of osteoporosis prevalence. Although femoral neck and total hip measurements were also included, future studies may benefit from prioritizing hip-based reference standards in degenerative cervical spine populations. Third, the osteoporosis subgroup was relatively small and markedly sex-imbalanced, with predominantly female patients, which limits the precision of osteoporosis-specific analyses and reduces the generalizability of sex-stratified findings, particularly in male osteoporotic patients. Fourth, medication history related to bone metabolism, including corticosteroids, antiresorptive therapy, anabolic agents, calcium supplementation, and vitamin D treatment, was not consistently available retrospectively and therefore could not be fully controlled. Fifth, BTMs were obtained at a single time point without fasting or timing standardization. Given the known biological variability of these markers and their sensitivity to circadian rhythm, nutritional status, medication exposure, and recent skeletal events, the observed associations should be interpreted as exploratory and hypothesis-generating. Although upper cervical HU measurements showed numerically higher AUC values than C-VBQ and Goutallier grading, formal pairwise ROC comparisons were not performed. Therefore, definitive superiority cannot be conclusively established.

## Conclusions

Upper cervical HU measurements, particularly at C2 and C3, demonstrated favorable discriminative ability for identifying reduced BMD in patients undergoing cervical spine surgery. Opportunistic C2 and C3 HU assessment may serve as a practical screening tool to identify patients who warrant further bone health evaluation, although prospective multicenter validation remains necessary before widespread clinical implementation.

## Funding

This work was supported by the 10.13039/501100001809National Natural Science Foundation of China (grant nos. 81972514, 82572811, 32371412, and 32071349), the Key Research and Development Program of Zhejiang Province (2025C02169), the Zhejiang Provincial Natural Science Foundation of China (grant nos. LY23H060008 and LY24C100001), and the National Health Commission Scientific Research Fund & Zhejiang Provincial Medical and Health Major Science and Technology Plan Project (WKJ-ZJ-2428).

## Ethical approval

Ethical approval for this study was obtained from the ethics committee of local hospital (No. 2024-0241) and adhered to the Declaration of Helsinki.

## Patient informed consent

All participants were informed about the research purpose and signed written consent.

## Data availability statement

Data presented in this study are available from the corresponding author upon request.

## Declaration of competing interests

The authors declare that they have no known competing financial interests or personal relationships that could have appeared to influence the work reported in this paper.
